# *In Vitro* and *In Vivo* Antitrypanosomal Activities of Methanol Extract of *Echinops kebericho* Roots

**DOI:** 10.1155/2020/8146756

**Published:** 2020-09-21

**Authors:** Debela Abdeta, Nigatu Kebede, Mirutse Giday, Getachew Terefe, Solomon Mequanente Abay

**Affiliations:** ^1^School of Veterinary Medicine, Wollega University, Nekemte, Ethiopia; ^2^School of Pharmacy, College of Health Sciences, Addis Ababa University, Addis Ababa, Ethiopia; ^3^Aklilu Lemma Institute of Pathobiology, Addis Ababa University, Addis Ababa, Ethiopia; ^4^Department of Veterinary Parasitology, Faculty of Veterinary Medicine and Agriculture, Addis Ababa University, Bishoftu, Ethiopia

## Abstract

Microbial resistance to the few conventional antitrypanosomal drugs, increasing resistance of vectors to insecticides, lack of effective vaccines, and adverse effects of the existing antitrypanosomal drugs justify the urgent need for effective, tolerable, and affordable drugs. We assessed antitrypanosomal effects of the hydromethanolic extract of *Echinops kebericho* Mesfin roots against *Trypanosoma congolense* field isolate using *in vitro* and *in vivo* techniques. Parasite load, packed cell volume (PCV), body weight, and rectal temperature in Swiss albino mice were assessed. This finding is part of the outcomes of drug discovery research for neglected tropical diseases. The extract arrested the motility of trypanosomes within 40 min at 4 and 2 mg/mL concentration, whereas in the untreated control, motility continued for more than 160 min. The extract also reduced parasitemia and prevented drop in PCV and body weight significantly (*p* < 0.05), as compared to control. Phytochemical analysis showed the presence of flavonoids, triterpenes, steroids, saponins, glycosides, tannins, and alkaloids. It is observed that this extract has activity against the parasite. Isolation and purification of specific compounds are required to identify hit compounds responsible for the antitrypanosomal activity of the studied medicinal plant.

## 1. Introduction

Current trypanosomiasis control relies on trypanocidal drugs, use of trypanotolerant cattle breeds, and control of the tsetse fly vector. The major strategy to control trypanosomiasis relied on the use of trypanocidal drugs, which is challenged by an increasing problem of resistance [1, 2]. The search for new chemical entities that are effective against trypanosomes, safe and affordable for disease-endemic countries, is rational to fight against trypanosomiasis [3]. To this effect, exploring natural products and synthetic sources are required to feed the pipeline of drug developments for trypanosomiasis control and elimination.

Plants are potential sources of new drugs due to the presence of countless number of secondary molecules that have pharmacological effects [4]. Exploring traditionally claimed medicinal plants for the biological activity gave humankind a number of antiprotozoal medications.

Validation of medicinal plants for their antitrypanosomal activities will guide the society for the best approach to employ their indigenous knowledge and at the same time provide hit compounds to feed future pipeline for antitrypanosomal drug development. *Echinops kebericho* (Mesfin) (Amharic vernacular name: *kebericho*), belonging to family Asteraceae/Compositae, endemic to Ethiopia, is an erect perennial herb or shrub [5]. Its varied medicinal applications are documented in the ancient medico-religious pharmacopoeia and are well-recognized by modern-day traditional professionals/specialists [6].

*Echinops kebericho* root is used for the treatment of animal trypanosomiasis in Ethiopia [7]. However, there is no laboratory-based evidence for the effectiveness and safety of this plant. The objective of this study was, therefore, to assess the *in vitro* and *in vivo* antitrypanosomal effects of the hydromethanolic extract of *E. kebericho* roots using field isolate of *T. congolense*, which is the most important cause of domestic animal trypanosomiasis. Experimental mice infection model has been selected given the provision of this model new insight in both human and animal trypanosomiasis [8, 9].

## 2. Method

### 2.1. Plant Collection and Authentication

Roots of *E. kebericho* were collected in November 2015 in Jimma Arjo Woreda of Eastern Wollega, Ethiopia. Leaves with flower specimen of the plant were identified and authenticated at Aklilu Lema Institute of Pathobiology (ALIPB), and the vouchers were deposited at the National Herbarium of Addis Ababa University with number DA 01.

### 2.2. Preparation of Plant Extracts

Air-dried powder of the plant material was macerated in an Erlenmeyer flask with 80% methanol at room temperature for a period of 72 hours. A total of 500 gm of dried plant material was used for extraction. A total of it was then filtered with gauze followed by Whatman filter paper (No. 1). The residue was remacerated once again to increase the yield. The filtrate was concentrated using a rotary evaporator at 40°C to remove methanol. Then, the concentrated filtrate was lyophilized to remove water to get dry power. The yield of hydromethanolic extract of *E. kebericho* roots was 11.7%.

### 2.3. Test Organism and Experimental Animals

*Trypanosoma congolense* was obtained from the Department of Veterinary Parasitology, Addis Ababa University, by infecting Swiss albino mice via intraperitoneal inoculation.

Swiss albino mice of either sex, weighing 30–35 g (age 10–12 weeks), were purchased from Ambo University (Ethiopia). They were fed with standard pellet, provided water *ad libitum*, and maintained at room temperature of 23–25°C with a relative humidity of 60–65%. The care and handling of animals were in accordance with internationally accepted guidelines for use of animals [10].

All applicable international, national, and/or institutional guidelines for the care and use of animals were followed. Ethical approval to conduct the research project was obtained from the Scientific and Ethics Committee of the Department of Pharmacology, School of Medicine, Addis Ababa University, with a letter dated November 18, 2015.

### 2.4. Experimental Procedures

#### 2.4.1. Evaluation of *In Vitro* Antitrypanosomal Activity

*In vitro* test was performed in triplicates to detect any motile trypanosomes in a 96-well microplate. Twenty microliters of blood containing about 16–32 organisms per field were mixed with 5 *μ*L of the test substance at concentrations of 2.5, 5, 10, and 20 mg/mL to produce test concentrations of 0.5, 1, 2, and 4.0 mg/mL, respectively.

Phosphate buffer saline (pH 7.2) and standard trypanocidal drug, diminazene aceturate (DA), were used to serve as untreated control and treated controls, respectively. The mixtures were incubated at 37°C for up to 3 h. During the period, motility of the parasites was checked in a 20-min interval under a microscope (×40 objective lens). In brief, about 2 *μ*L of test mixtures was placed on a microscope slide and covered with cover slips, and the parasites were observed for reduced motility or complete cessation of motility.

#### 2.4.2. Evaluation of *In Vivo* Antitrypanosomal Activity

Thirty mice of either sex were randomly grouped into five (I–V) groups containing 6 animals per group. They were intraperitoneally infected with 0.2 mL of *T. congolense* (5 × 10^5^ parasites/mL) suspension. Groups I and II were administered only 0.3 mL of distilled water per oral administration to serve as negative control and 3.35 mg/kg of DA in distilled water per oral administration to serve as positive control, while groups III, IV, and V were administered with the extract dissolved in distilled water (the solvent for control group) at daily doses of 100, 200, and 400 mg/kg body weight, respectively, for 7 consecutive days per oral administration from the 10^th^ day of parasite inoculation. Parasitemia and packed cell volume (PCV) were observed every 4 days for 21 days, while body weight and rectal temperature was monitored every 2 days [11].

#### 2.4.3. Determination of Parasitemia

On the tenth day after infection and every four days, the parasitemia level of mice was checked. Parasitemia was monitored by examining blood drawn from the tail of mice under the microscope at ×400 magnification using the “Rapid Matching” method of Herbert and Lumsden [12]. Monitoring of parasitemia was performed every four days until the 21^st^ day posttreatment initiation [13, 14].

#### 2.4.4. Determination of Packed Cell Volume

PCV was determined using a microhematocrit centrifuge and microhematocrit tube reader. PCV was monitored on the day of treatment initiation and every 4 days until 21^st^ day of posttreatment initiation [15, 16].

#### 2.4.5. Determination of Body Weight

Body weight of experimental animals was recorded on the day of parasite challenge, day of treatment initiation, and everyday for 21 days [17].

#### 2.4.6. Determination of Rectal Temperature

Rectal temperature was measured using a digital rectal thermometer (Mettler Toledo, Switzerland) on the day of parasite inoculation, day of treatment commencement, and everyday thereafter for 21 days [15].

#### 2.4.7. Phytochemical Screening for Secondary Metabolites

Standard screening tests of the extract were carried out for secondary metabolites according to the methods described in the literature [18–23].

### 2.5. Statistical Analysis

Data were presented as mean ± SEM and analyzed using Statistical Package for Social Sciences version 20. Analysis of variance was employed to test statistical difference within all groups followed by Tukey's test for significance test between two groups. *p* values less than 0.05 were considered statistically significant.

## 3. Results

### 3.1. Experimental Animals Follow-Up

**Figure 1:**
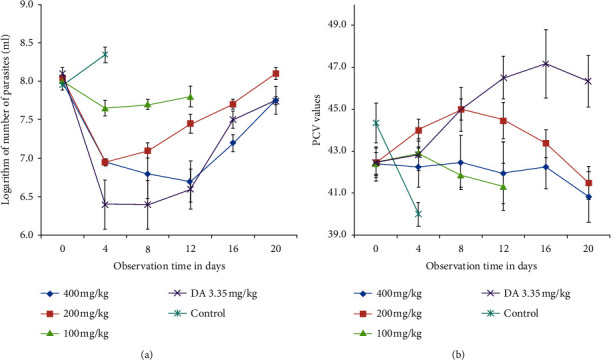
Effect of *E. kebericho* root extract. (a) Effect on parasitemia of *T. congolense*-infected mice. (b) Effect on PCV of *T. congolense*-infected mice. Values are expressed as mean ± SEM, day 0 = 10^th^ day after infected blood inoculation, DA = diminazene aceturate.

**Figure 2:**
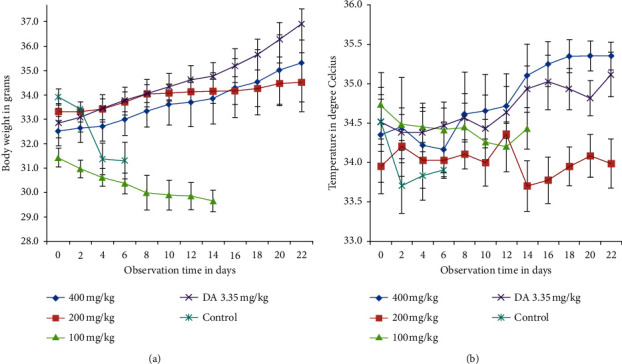
Effect of *E. kebericho* root extract. (a) Effect on body weight of *T. congolense*-infected mice. (b) Effect on rectal temperature of *T. congolense*-infected mice. Values are expressed as mean ± SEM, *n* = 6, day 0 = 10^th^ day after infected blood inoculation, DA = diminazene aceturate.

Parasite load, body temperature, and weight of animals were recorded. When the parasite load increased and confirmed that experimental animals could not survive due to infection, inhalation anesthetic in a transparent euthanasia chamber was used for humane euthanasia of experimental animals. Data set collected from solvent control and low-dose-extract-treated groups were only for few days (Figures 1 and 2) due to rapid increment of parasite load.

### 3.2. *In Vitro* Antitrypanosomal Activity

Hydromethanol extracts of *E. kebericho* roots ceased motility of the trypanosomes within 40 min at 4 and 2 mg/mL concentration. At 0.5 mg/mL of the hydromethanol extract of *E. kebericho* roots, the motility was maintained for 80 min after which motility of the parasite is completely ceased. The motility of parasites ceased at 60 minutes for the *E. kebericho* roots extract at a dose of 1 mg/mL (Table 1).

### 3.3. Effect on Parasitemia of *T. congolense*-Infected Mice

Parasitemia level was assessed at day 4 posttreatment in all thirty experimental animals randomly assigned to different groups (30/30). All doses of the extract including the standard drug suppressed parasitemia at day 4 posttreatment (*p* < 0.05). Treatment with extracts at 200 mg/kg and 400 mg/kg and DA 3.35 mg/kg showed statistically significant (*p* < 0.05) reduction in parasitemia on day 8 to day 12 posttreatments compared to 100 mg/kg body weight (Figure 1(a)).

### 3.4. Effect on Packed Cell Volume of *T. congolense*-Infected Mice

While the mean PCV in the untreated control group continued to decrease until all the animals died due to infection, 200 mg/kg and 400 mg/kg of the extract and DA 3.35 mg/kg show an increase in mean PCV values from day 0 to day 12 treatment follow-up. PCV measurement in blood of infected mice treated with *E. kebericho* at a dose of 200 mg/kg and 400 mg/kg and DA 3.35 mg/kg showed statistically significant (*p* < 0.05) improvement compared with those untreated control on day 4 posttreatment initiation (Figure 1(b)).

### 3.5. Effect on Body Weight of *T. congolense*-Infected Mice

There are statistically significant (*p* < 0.05) body weight changes in 200 mg/kg-, 400 mg/kg-, and DA 3.35 mg/kg-treated groups compared with 100 mg/kg through day 4 to day 6 posttreatment initiation and with untreated control on day 4 posttreatment (Figure 2(a)).

### 3.6. Effects on Rectal Temperature of *T. congolense*-Infected Mice

The rectal temperatures of the animals were fluctuating throughout the experiment. There is no observed difference throughout the follow-up period (Figure 2(b)).

#### 3.6.1. Phytochemical Screening

Phytochemical screening revealed the presence of saponins, tannins, phenol, terpenes, flavonoids, glycosides, and alkaloids.

## 4. Discussion

In the present study, antitrypanosomal activities of the *E. kebericho* roots suggested that the extract could contain trypanocidal constituents that are active in the *in vitro* and *in vivo* environments. Parasites' motility constitutes a relatively reliable indicator of viability of most trypanosomes [24], and a complete elimination or reduction in the motility of trypanosomes when compared to the control could be taken as index of trypanocidal activity [25].

*In vivo* assessment of the extract revealed a marked suppression of parasite load at 200 and 400 mg/kg compared to group treated with vehicle control even though the extract failed to clear the parasite. Further investigation is required to see whether the extract will have an improved effect or not when administered by injection to minimize the negative impact of limited bioavailability from the gut.

The mean PCV in the untreated control group continued to decrease until all the animals in the group died due to infection, while in treated groups, the value shows normal range. The decrease in PCV value for untreated control may be due to anemia which is the most outstanding clinical and laboratory feature of African trypanosomiasis [26].

The present study showed that the *E. kebericho* extract contains secondary metabolites: saponins, tannins, phenol, terpenes, flavonoids, glycosides, and alkaloids. The responsible active components were yet to be isolated. Previous studies showed that flavonoids are effective antitrypanosomal substances against different trypanosome species [27]. Phenolics and polyphenols have also been reported to have antitrypanosomal activity by inhibiting the trypanosome alternative oxidase [28]. Alkaloids affect trypanosomes by DNA intercalation in combination with the inhibition of protein synthesis [27]. The *in vitro* and *in vivo* activities of the *E. kebericho* extract in the present study might be contributed by multiple secondary metabolites.

The present study also demonstrated that the extract is tolerable since no treatment-related signs of toxicity were noticed in the animals throughout the observation period. Per oral administration of the hydromethanolic extract of *E. kebericho* root extract produced neither significant toxic signs nor death during the observation period of 14 days after a single administration of 2000 mg/kg with oral median lethal dose greater than 2000 mg/kg in mice. Another study showed that the hydromethanolic extract of *E. kebericho* roots (up to a dose of 5,000 mg/kg) did not produce a sign of toxicity [29].

In conclusion, the present study provides evidence to the antitrypanosomal activity of the hydromethanolic extract of *E. kebericho* roots and validates the traditional practice of Ethiopian community to control trypanosomiasis. Further *in vitro* and *in vivo* activities of the extract on other species of trypanosome are recommended. In addition, the responsible compound(s) for activity shall be characterized to identify hits and develop lead compounds.

## Figures and Tables

**Table 1:** *In vitro* activity of hydromethanolic extract of *E. kebericho* roots.

Duration (min)	*E. kebericho* extract (motility)				DA 3.35 mg/ml (motility)	Control (motility)
	0.5 mg/mL	1 mg/mL	2 mg/mL	4 mg/mL		
0	+	+	+	+	+	+
20	+	+	+	+	−	+
40	+	+	−	−	−	+
60	+	+	−	−	−	+
80	+	−	−	−	−	+
100	−	−	−	−	−	+
120	−	−	−	−	−	+
140	−	−	−	−	−	+
160	−	−	−	−	−	+
180	−	−	−	−	−	−
200	−	−	−	−	−	−

## Data Availability

The data used to support the findings of this study are available from the corresponding author upon request.

## Abbreviations

PCV: Packed cell volume
ALIPB: Aklilu Lemma Institute of Pathobiology
DA: Diminazene aceturate
OECD: Organization for Economic Co-operation and Development.
